# A Machine Learning Approach for the Association of ki-67 Scoring with Prognostic Factors

**DOI:** 10.1155/2018/1912438

**Published:** 2018-08-07

**Authors:** E. Dirican, E. Kiliç

**Affiliations:** ^1^Biostatistics, Faculty of Medicine, Mustafa Kemal University, Hatay 31000, Turkey; ^2^General Surgery, Faculty of Medicine, Mustafa Kemal University, Hatay 31000, Turkey

## Abstract

ki-67 score is a solid tumor proliferation marker being associated with the prognosis of breast carcinoma and its response to neoadjuvant chemotherapy. In the present study, we aimed to investigate the way of clustering of prognostic factors by ki-67 score using a machine learning approach and multiple correspondence analysis. In this study, 223 patients with breast carcinoma were analyzed using the random forest method for classification of prognostic factors according to ki-67 groups (<14% and >14%). Also the relationship between subgroups of prognostic factors and ki-67 scores was examined by multiple correspondence analysis. There was a clustering of molecular classification LA, 0-3 metastatic lymph node, age <50, absence of LVI, T1 tumor size with ki-67 <14% and grade III, 10 or more metastatic lymph nodes, and presence of LVI and molecular classification LB, age >50, and T3-T4 tumor size categories with ki-67 >14%. The fact that the low scores of ki-67 correlate with early stage diseases and high scores with advanced disease suggests that 14% threshold value is crucial for ki-67 score.

## 1. Introduction

Machine learning investigates how computers can learn (or improve their performance) based on available data. A main research area for computer programs is to automatically learn to recognize complex patterns and make intelligent decisions based on available data [1]. Random forest (RF) is a supervised machine learning technique and a combination of tree predictors in which each tree depends on the values of a random vector sampled independently and with the same distribution for all the trees in the forest [2].

ki-67 score is the core protein expressed at G1, S, G2, and M phases of tumor cells and a solid tumor proliferation marker being associated with prognosis of breast carcinoma (BC) and its response to neoadjuvant chemotherapy [3]. A threshold value of 14% is determinant for the identification of molecular subtypes BCs (MSBC). Chemotherapy response and progression of MSBCs differ [4, 5].

The molecular subtypes of breast cancer (MSBC) are defined based on the expression of ki-67, estrogen receptor (ER), progesterone receptor (PR), and human epidermal growth factor receptor-2 (HER-2) [6].

In luminal A (LA), ER and/or PR is positive, HER-2 is negative, and the proliferation index is low. In luminal B (LB), tumors are of high grade and may be PR+ or PR- or HER2+ or HER2-. If they are HER2-, they can be distinguished from LA by ki-67 score being >14% [3]. HER-2: HER-2 gene expression is high; however, ER and PR are negative and they are of high grade with ki-67 score of >14% [6]. Typically, triple negative breast carcinoma (TNBC) is the type lacking ER and PR with overexpression of HER2. Compared to other subtypes, TNBC tumors are usually larger [7, 8] and they are associated with 2.5-fold more metastasis within five years after diagnosis [8].

Lymphovascular invasion (LVI) is present in one-third of BCs. As a single indicator of adjuvant chemotherapy [7], LVI is associated with increased lymph node metastasis and the risk of progression to systemic disease [9, 10]. It is a negative effective factor in survival for relapse and survival in node-negative patients [11].

Age is a prognostic factor in BC and varies by geographical region or demographics. In regions with young populations such as Asia, Africa, and Turkey, BCs are more frequent under the age of 40, and these tumors are found at further stages compared to the Western societies [12]. The presence of axillary lymph node (LN) is one of the most important factors in prognosis estimation for the patients. Metastatic axillary lymph node ratio (mALNR) is known as an important factor in survival for BC [13]. In general, high mALNR indicates poor prognosis [14, 15]. Spread of cancer cells to regional LNs is the most important prognostic factor and, assessing the status of axillary lymph nodes (ALNs) is important for the prediction of long-term survival in BC [16, 17]. In developed countries, histologically node-negative breast carcinoma (HNNBC) accounts for two-thirds of invasive BC [18]. Histologically node-negative BC patients usually have a good prognosis [18, 19].

Histopathological grade is a special prognostic factor. Some recent studies have confirmed the importance of histopathological grading of BC as a predictive and effective factor in survival. Grade 2 and 3 BCs have poorer prognosis [20, 21]. Tumor size (TS) is an independent prognostic factor independent in TNM staging system and it shows a good correlation with nodal metastasis incidence, relapse risk, and survival [22, 23]. In the present study, we aimed to investigate the way of clustering of prognostic factors by ki-67 score using a machine learning approach and multiple correspondence analysis (MCA).

## 2. Materials and Methods

Patients with BC treated at Mustafa Kemal University, Faculty of Medicine, Research Hospital, General Surgery Clinic, between January 2014 and December 2017, were analyzed. The study was designed retrospectively and conducted at Mustafa Kemal University, Medical School General Surgery Department, following the approval of Mustafa Kemal University Clinical Research Ethics Committee (approval date, March 22,2018; 75).

Data regarding the prognostic factors including patient's age, body mass index (BMI), TS (cm), ki-67 score (%), ER, PR, c-erb-2 receptor status, molecular classification (MC) (LA, LB, Her-2 and TNBC) data, histopathological diagnosis, nuclear grade status (Modified Bloom Richardson), mALNscount (pN1, pN2, pN3), LVI, and the methods of operation were recorded. The way of clustering of ki-67 scores with prognostic variables was examined.

It was included in the range of 18 to 70 years of age in the study, patients with distant metastasis and morbid obesity (BMI ≥ 40) were excluded. Most of patients (86%) had invasive ductal carcinoma. In the case of a sufficient number of patients with a molecular class of TNBC, it was thought that TNBC could cluster with ki-67 classes.

Also, as number of LN, BMI, perivascular invasion (PVI), and histopathological type variables reduced the total inertia (58%) and caused ambiguity for variable clustering, they were excluded from the analysis. Furthermore, as the effect of surgical type variable on ki-67 classification is neglected, it was excluded from the MCA.

Univariate analyses, RF machine learning classification algorithm, and MCA statistical methods were used for data evaluation. For 16 missing values among different prognostic variables in data set, “rfimpute” RF value imputation algorithm was used. RF is a classification method involving a voting method. It is comprised by many decision trees [2]. Decision trees are independent from each other and formed by samples withdrawn from the data set using bootstrap method.

*X* input vector: (*X*), ${\hat{C}}_{rf}^{B}=majority\;\;vote\{\hat{C}\left(X\right){\}}_{1}^{B}$ where ${\hat{C}}_{b}\left(X\right)$ is the class prediction of the *b*_th_RF tree. During RF classification procedure, relative significance of different variables is also evaluated [24]. This study took the decrease in GINI index into consideration to evaluate the significance of each variable. The GINI index measures the impurity or inequality level of a sample assigned to a node [25].

Supervised machine learning approach was used in analyzing relationship on between as label ki-67 groups and input variables (MC, LVI, age, number of mLN, nuclear grade, TS, number of LNs, BMI, PVI, surgical type, and histopathological type). Thus in this study, classificability of prognostic variables by ki-67 groups (<14% and >14%) was analyzed using RF method. In the train set, 10-fold cross-validation method was applied for the parametric optimization of machine learning algorithm. Test set was used to determine the accuracy of the learned model. For the evaluation of model performance, the Receiver Operating Characteristics (ROC) curve and area under the curve (AUC) were calculated.

In the correspondence analysis, having no distribution assumption except the assumption that the frequencies in the cross table are positive numbers, the correspondence analysis aims to graphically demonstrate the association between the rows and columns in cross tables and develop simple factors by providing this demonstration [26]. In our study, we used MCA to reveal the association of ki-67 with prognostic factors.

## 3. Results

A total of 223 patients with breast carcinoma were included in this study. A total of 74 cases (32%) had a ki-67 score of <14% with a mean age of 52.5 ± 12.14 years. A total of 149 cases (66.8%) had a ki-67 score of >14% with a mean age of 50.75 ± 11.95 years. As in general terms, our study was built on the association of ki-67 scoring with variables qualified as prognostic factor for BC; the results of RF method were taken into account (Age, Number of mLNs, Histopathological Type and BMI, p = 0.742, p = 0.234, p = 0.403 and p = 0.386, respectively) rather than the nonsignificant *p* values in Table 1; as significance control for the variables was also performed using the applied RF algorithm. Many cases had the histological type of invasive ductal carcinoma (86%) and the highest grade was Grade II (51.6%). By BMI groups, there were no underweight patients and most of the patients (56.9%) were in the obese group. The distribution of nonmetastatic lymph node count (nmLN) varies by ki-67 groups and classes (p = 0.07).

**Figure 1:**
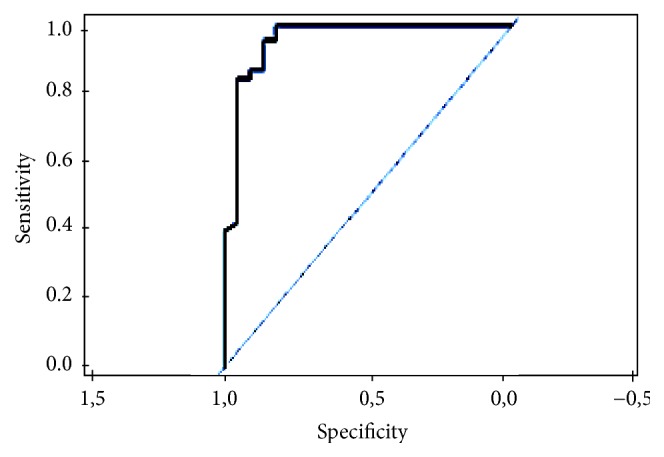
ROC Curve for RF performance.

Using m_try_ = 3 as number of discriminant variables in decision trees and ntree = 100 as number of used trees, RFClassification Algorithm was applied to the data set involving 223 cases. Using all these arguments, the obtained accuracy was 0.91. For the evaluation of the performance of the obtained model, the ROC curve and AUC were calculated. Using the analysis, AUC was found at 0.95 (Figure 1).

**Figure 2:**
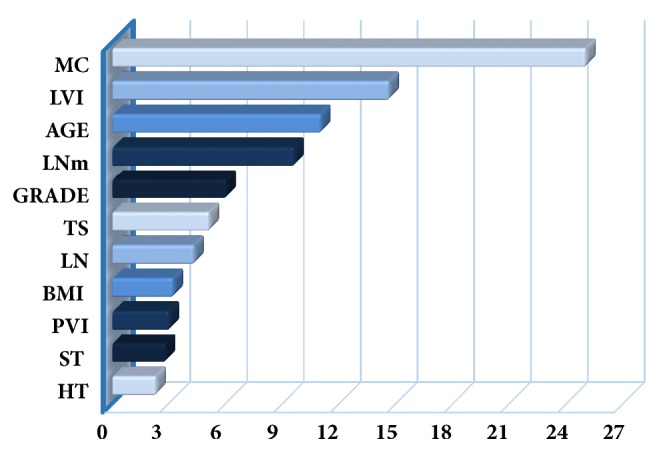
Mean decrease GINI.* MC: molecular classification, LVI: lymphovascular invasion, LNm: number of metastatic lymph nodes, TS: tumor size, LN: number of metastatic/nonmetastatic lymph nodes, BMI: body mass index, PVI: perivascular invasion, ST: surgical type/procedure, *and* HT: hypertension*.

According to the association of ki-67 with the prognostic variables for breast carcinoma, the variables with high and low significance are shown in Figure 2. Figure 2 was designed based on the mean decrease in GINI. According to this figure, the variable with the most contribution to ki-67 classification is MC (25), followed by LVI (14.6), age (11), number of mLN (9.6), nuclear grade (6), TS (5.1), number of LNs (4.3), BMI (3.2), PVI (3), and surgical type (2.8) in descending order with the variable in the GINI index with the least contribution to the classification being histopathological type (2.3).

MCA was performed to determine the association of ki-67 proliferation with other variables. For this analysis, MC, LVI, age, number of mLNs, nuclear grade, and TS variables were taken from Figure 2.

**Figure 3:**
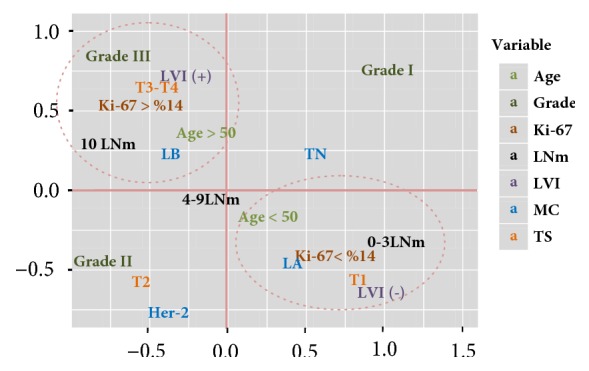
MCA plot of variables association with ki-67.* LNm: number of metastatic lymph nodes, LVI: lymphovascular invasion, MC: molecular classification, TS: tumor size, LA/LB: luminal A/B, *and* Her-2: Her-2 breast carcinoma.*

The association of variables in two dimensions in MCA was explained by 76.303% (Dim. 1 + Dim. 2 = 41.093 + 35.209 = 76.303). According to this analysis, in case of ki-67 tumor proliferation over 14%, clustering for grade III, 10 or more mLNs, presence of LVI, LB, age over 50 years, and T3-T4 was observed. In cases of ki-67 tumor proliferation below 14%, clustering for LA, 0-3 mLNs, absence of LVI, age below 50 years, and T1 was observed. However, none of the ki-67 groups showed clustering for Grades I and II, T2, Her 2, and TNBC, 4-9 mLNs (Figure 3).

## 4. Discussion

### 4.1. Molecular Subtype Breast Carcinoma and ki-67 Scoring

ki-67 is the core protein expressed at G1, S, G2, and M phases of tumor cells and a solid tumor proliferation marker [3]. In this study, ki-67 groups (<14%/>14%) and MSBC were determined according to St. Gallen consensus [4]. 74 (33.2%) cases had ki-67 score of < 14%, and 149 cases (66.8%) had >14% (p = 0.001).

#### 4.1.1. Luminal A, Luminal B, and Her-2.

While normally ki-67 >14% class should not have LA, as imputation was performed for the parameters with missing data using “rfinput” command in the “RandomForest” package in R software, one LA was present in this section. To avoid that these missing data decrease the safety of the analysis, even though they are very few, this random procedure was not interfered. 37.8% of the cases with ki-67 score of <14% and 75.2% of the cases with >14% were LB. In this study, while there were 6 patients (8.2%) with Her-2 molecular type with ki-67 score of <14% and 27 patients (18.1%) with Her-2 molecular type with ki-67 score of >14%. Molecular subtyping was detected to be the most important factor decreasing the mean GINI index and, consistent with the literature, LB showed clustering with ki-67 score of >14% and LA with ki-67 of <14%. Her-2 did not show clustering in neither of the groups.

#### 4.1.2. Triple Negative Breast Cancer

ki-67 score of <14% was detected in 3 (4%) cases and ki-67 of >14% in 9 (6%) cases with TNBC. However, it did not show clustering with ki-67 scores (see Figure 3). The reason that TNBC did not show clustering with any of the subgroups of prognostic factors in MCA is the insufficient number of TNBC in data set.

#### 4.1.3. Lymphovascular Invasion 

In this study, LVI distribution was detected as 38 cases (51.4%) for ki-67 score of <14% and 117 cases (78.5%) for ki-67 of >14%. LVI showed significant clustering with the other prognostic variables and ki-67 groups (ki-67>14%--LVI(+) and ki-67<14%--LV(-)). It is based on 14% of the ki-67 score which is similar to our study [27] reporting that patients with high ki-67 expression had significantly high rates of LVI. Coexistence of LVI and ki-67 score of >14% was considered to indicate poor prognosis and systemic disease [28].

#### 4.1.4. Metastatic/Nonmetastatic Lymph Node Count

Axillary lymph node metastasis is an important biological feature of BC, and it leads to poor prognosis and death [29]. LVI is a powerful predictor of axillary metastasis [30]. mALNs are grouped as 0-3, 4-9, and ≥10; mLNs and nmLNs were detected to be comparable for both groups [31]. ≥10 mALN showed clustering for ki-67 group of >14% and 0-3 mALN for ki-67 of <14%. As aforementioned ki-67 score of >14% showed clustering with LVI (+) and ≥10 mALN [29] (see Figure 3). nmALN was detected to be 2.39 ± 4.62 in ki-67 group of <14% and 3.74 ± 6.3 in ki-67 group of >14% (p = 0.07). As the mean GINI index was low, it was excluded from the MCA.

#### 4.1.5. Nuclear Grade

The histopathological grade was determined using the modified Scarff-Bloom-Richardson grading system (Nottingham Combined Histological Grade) [32]. When the groups were assessed for nuclear grade, grade 2 was significantly more in both groups (p = 0.001). Clustering was observed for grade III group with ki-67 class of >14%, and no clustering was observed for Grades I and II with any of ki-67 scores (Figure 3). This was considered to develop due to grade and ki-67 scores increased secondarily to nuclear proliferation developed at G1, S, G2, and M phases [3]. Consistent with previous studies, nuclear grade and ki-67 were found to be of positive correlation between scores [33].

#### 4.1.6. Age

In the present study, the number of 50-year-old or younger patients was more in both ki-67 score groups (p = 0.742). The association of age groups with ki-67 score classes was evaluated, and clustering was observed for ki-67 of >14% with 50-year-old or older patients and for ki- 67 of <14% with patients younger than 50 years (Figure 3).

The variable of age and ki-67> 14% scores in study showed negative correlation [34] in contrast to our study; the positive correlation was found. This situation is thought to be caused by the difference of the population in which the sample is drawn.

#### 4.1.7. Tumor Size

Although, in many studies, there was no correlation between TS size and ki-67 score [35–37] in the present study, clustering was observed between T3/4 and ki-67 class of >14% and T1 and ki-67 of <14%. However, T2 did not show clustering with any of ki-67 classes (Figure 3). For the tumors at the same T stage, the risk of progression to advanced stage disease increases with the increasing size [31]. TS was considered to increase secondary to progression development with high ki-67 score.

Successful results were obtained in the study using [38] “k-Means clustering” classification method. However, as “k-Means clustering” method lays equal weight to each attribute during the classification, it may cause predicaments for unrelated attributes. Hence, in our study which also examines the association of the data, there are also attributes with no association with ki-67 scoring. Along with the aforementioned parameters, RF method was applied using R 3.3.3 program and the accuracy was found 91%. For validity of the results, the ROC analysis was conducted to evaluate the performance in our study, and AUC was found 0.95.RF method, it was preferred because of its advantages such as possibility of evaluating the relative importance of the variables in classification, the ability to identify variable interactions, and the short operation time.

As a graphical method is used during the analysis of the association between the categories of variables in MCA, it is considered to be more successful than the clustering analysis. In the study examining the prognostic factors correlated with ki-67 [35], the association was examined using univariate analysis such as ANOVA and chi-square test.

In our study of which the majority of data is categorical, a type of multivariate analysis MCA which also takes the visual dimension of the association into account was used. For MCA, the variance for two dimensions was found 76.30%. Among the variables contributing to inertia, the association of grade, mLN, MC, LVI, age, and TS was examined.

## 5. Conclusion

Luminal B, nuclear grade III, age ≥50 years, LVI (+), number of mLNs ≥10, tumor size T3/4, and ki-67 > 14% clusters were observed in the analysis of the relation between ki-67 threshold value and prognostic factors. Luminal A, age <50 years, LVI (-), number of mLNs 0-3, and tumor T1 were clustered with ki-67 < 14% score. The fact that the low scores of ki-67 correlate with early stage diseases and high scores with advanced disease suggests that 14% threshold value is crucial for ki-67 score.

## Figures and Tables

**Table 1:** Patient characteristics (discrete data are given as numbers, continuous as the mean ± standard deviation) (n = 223).

**Variable**		**Ki-67 ≤ **14%	** Ki-67 > **14%	**p**
**Ki-67 Proliferation**		74 (33.2)	149 (66.8)	0.001


**Molecular Classification**				
	LA	37 (50)	1 (0.7)!	0.001
	LB	28 (37.8)	112 (75.2)	
	Her-2	6 (8.2)	27 (18.1)	
	TN	3 (4)	9 (6)	

**Lymphovascular Invasion**				
	-	36 (48.6)	32 (21.5)	0.001
	+	38 (51.4)	117 (78.5)	

**Age**				
	Age <50	38 (51.4)	80 (53.7)	0.742
	Age >50	36 (48.6)	69 (46.3)	

**Number of Metastatic Lymph Nodes**				
	0-3 LNm	27 (36.5)	38 25.5)	0.234
	4-9 LNm	8 (10.8)	18 (12.1)	
	10 LNm	39 (52.7)	93 (62.4)	

**Nuclear Grade**				
	Grade I	26 (35.1)	16 (10.7)	0.001
	Grade II	33 (44.6)	82 (55)	
	Grade III	15 (20.3)	51 (34.2)	

**Tumor Size**				
	T1	32 (43.2)	41 (27.5)	0.01
	T2	38 (51.4)	82 (55)	
	T3 and T4*∗∗*	4 (5.4)	26 (17.4)	

**Body Mass Index**				
	18.5-24.9	7 (9.5)	18 (12.1)	0,386
	25-29.9	28 (37.8)	43 (28.9)	
	30^+^	39 (52.7)	88 (59.1)	

**Surgical Type**				
	Mastectomy	55 (74.3)	77 (51.7)	0,001
	Segmental Mastectomy	19 (25.7)	72 (48.3)	

**Histopathological Type**				
	IDC	61 (82.4)	131 (87.9)	0,403
	ILC	6 (8.1)	6 (4)	
	Other*∗*	7 (9.5)	12 (8.1)	

**Perivascular Invasion**				
	-	52 (70.3)	36 (24.2)	0.01
	+	22 (29.7)	113 (75.8)	

**Number of Lymph Nodes**				
		2.39 ± 4.62	3.74 ± 6.3	0.07

*∗*: 6 mucinous carcinomas, 7 DCIS, 6 neuroendocrine carcinomas. **!:** “rfinput” imputation. **Molecular classification:** LA: luminal A, LB: luminal B, and TNBC: triple negative breast carcinoma; **BMI:** underweight <18.50, normal range 18.50-24.99, overweight ≥ 25.00, and obese ≥ 30.00.

## Data Availability

Access to data is restricted, because the institution from which the data is received does not allow the sharing of data with third parties in terms of patient privacy.

## References

[1] Han J., Kamber M., Pei J. (2012). *Data Mining Concepts and Techniques*.

[2] Breiman L. (2001). Random forests. *Machine Learning*.

[3] Jones R. L., Salter J., A'Hern R. (2009). The prognostic significance of Ki67 before and after neoadjuvant chemotherapy in breast cancer. *Breast Cancer Research and Treatment*.

[4] Goldhirsch A., Wood W. C., Coates A. S., Gelber R. D., Thürlimann B., Senn H.-J. (2011). Strategies for subtypes-dealing with the diversity of breast cancer: highlights of the St Gallen international expert consensus on the primary therapy of early breast cancer 2011. *Annals of Oncology*.

[5] Goldhirsch A., Winer E. P., Coates A. S., Gelber R. D., Piccart-Gebhart M., Thürlimann B. B. Personalizing the treatment of women with early breast cancer: highlights of the St Gallen International Expert Consensus on the Primary Therapy of Early Breast Cancer 2013. *Annals of Oncology*.

[6] Peppercorn J., Perou C. M., Carey L. A. (2008). Molecular subtypes in breast cancer evaluation and management: Divide and conquer. *Cancer Investigation*.

[7] Lehmann B. D., Bauer J. A., Chen X. (2011). Identification of human triple-negative breast cancer subtypes and preclinical models for selection of targeted therapies. *The Journal of Clinical Investigation*.

[8] Dent R., Trudeau M., Pritchard K. I. (2007). Triple-negative breast cancer: clinical features and patterns of recurrence. *Clinical Cancer Research*.

[9] Cornwell L. B., McMasters K. M., Chagpar A. B. (2011). The impact of lymphovascular invasion on lymph node status in patients with breast cancer. *The American Surgeon*.

[10] Wong J., O'Neill A., Recht A. (1998). The relationship between lymphatic vessel invasion, tumor size and pathologic nodal status: Can we predict who can avoid a third field in the absence of axillary dissection. *International Journal of Radiation Oncology • Biology • Physics*.

[11] Lee A. H. S., Pinder S. E., Macmillan R. D. (2006). Prognostic value of lymphovascular invasion in women with lymph node negative invasive breast carcinoma. *European Journal of Cancer*.

[12] Agarwal G., Pradeep P. V., Aggarwal V., Yip C.-H., Cheung P. S. Y. (2007). Spectrum of breast cancer in Asian women. *World Journal of Surgery*.

[13] Woodward W. A., Vinh-Hung V., Ueno N. T. (2006). Prognostic value of nodal ratios in node-positive breast cancer. *Journal of Clinical Oncology*.

[14] Vinh-Hung V., Verkooijen H. M., Fioretta G. (2009). Lymph node ratio as an alternative to pN staging in node-positive breast cancer. *Journal of Clinical Oncology*.

[15] Ahn S. H., Kim H. J., Lee J. W. (2011). Lymph node ratio and pN staging in patients with node-positive breast cancer: A report from the Korean breast cancer society. *Breast Cancer Research and Treatment*.

[16] Gujam F. J. A., Going J. J., Edwards J., Mohammed Z. M. A., McMillan D. C. (2014). The role of lymphatic and blood vessel invasion in predicting survival and methods of detection in patients with primary operable breast cancer. *Critical Review in Oncology/Hematology*.

[17] Thompson A. M. (2012). New standards of care in the management of the axilla. *Current Opinion in Oncology*.

[18] Harbeck N., Thomssen C. (2011). A new look at node-negative breast cancer.. *The Oncologist*.

[19] Habel L. A., Shak S., Jacobs M. K. (2006). A population-based study of tumor gene expression and risk of breast cancer death among lymp node-negative patients. *Breast Cancer Research*.

[20] Engstrøm M. J., Opdahl S., Hagen A. I. (2013). Molecular subtypes, histopathological grade and survival in a historic cohort of breast cancer patients. *Breast Cancer Research and Treatment*.

[21] Luangdilok S., Samarnthai N., Korphaisarn K. (2014). Association between pathological complete response and outcome following neoadjuvant chemotherapy in locally advanced breast cancer patients. *Journal of Breast Cancer*.

[22] Tavasolli F. A., Devilee P. (2003). *World Health Organisation Classification of Tumours. Pathology and Genetics of the Breast and Female Genital Organs*.

[23] Tavasolli F. A. (1999). *Pathology of the Breast*.

[24] Gislason P. O., Benediktsson J. A., Sveinsson J. R. (2006). Random forests for land cover classification. *Pattern Recognition Letters*.

[25] Zhang C., Ma Y. (2012). *Ensemble Machine Learning: Methods and Applications*.

[26] Alpar R. (2013). *Uygulamali çok değişkenli istatistiksel yöntemler*.

[27] Shen S., Xiao G., Du R., Hu N., Xia X., Zhou H. (2018). Predictors of lymphovascular invasion identified from pathological factors in Chinese patients with breast cancer. *Oncotarget *.

[28] Ermiah E., Buhmeida A., Abdalla F. (2012). Prognostic value of proliferation markers: Immunohistochemical Ki-67 expression and cytometric S-phase fraction of women with breast cancer in Libya. *Journal of Cancer*.

[29] Donegan W. L. (1997). Tumor-related prognostic factors for breast cancer. *CA: A Cancer Journal for Clinicians*.

[30] Bevilacqua J. L. B., Kattan M. W., Fey J. V., Cody H. S., Borgen P. I., Van Zee K. J. (2007). Doctor, what are my chances of having a positive sentinel node? A validated nomogram for risk estimation. *Journal of Clinical Oncology*.

[31] Coburn N. G., Chung M. A., Fulton J., Cady B. (2017). Decreased Breast Cancer Tumor Size, Stage, and Mortality in Rhode Island: An Example of a Well-Screened Population. *Cancer Control*.

[32] Elston C. W., Ellis I. O. (2002). Pathological prognostic factors in breast cancer. I. The value of histological grade in breast cancer: experience from a large study with long-term follow-up. C. W. Elston & I. O. Ellis. *Histopathology* 1991; 19; 403–410. *Histopathology*.

[33] Inwald E. C., Klinkhammer-Schalke M., Hofstädter F. (2013). Ki-67 is a prognostic parameter in breast cancer patients: Results of a large population-based cohort of a cancer registry. *Breast Cancer Research and Treatment*.

[34] Klauber-DeMore N. (2005). Tumor biology of breast cancer in young women. *Breast Disease*.

[35] Madani S.-H., Payandeh M., Sadeghi M., Motamed H., Sadeghi E. (2016). The correlation between Ki-67 with other prognostic factors in breast cancer: A study in Iranian patients. *Indian Journal of Medical and Paediatric Oncology*.

[36] Nahed A. S., Shaimaa M. Y. (2016). Ki-67 as a prognostic marker according to breast cancer molecular subtype. *Cancer Biology & Medicine*.

[37] Haroon S., Hashmi A. A., Khurshid A. (2013). Ki67 Index in Breast Cancer: Correlation with Other Prognostic Markers and Potential in Pakistani Patients. *Asian Pacific Journal of Cancer Prevention*.

[38] Lopez X. M., Debeir O., Maris C. (2012). Clustering methods applied in the detection of Ki67 hot-spots in whole tumor slide images: an efficient way to characterize heterogeneous tissue-based biomarkers. *Cytometry Part A*.

